# Schema validation and evaluation framework for extracted schemas in JSON databases

**DOI:** 10.1038/s41598-026-45554-6

**Published:** 2026-03-27

**Authors:** Saad Belefqih, Mohammed Barchane, Ahmed Zellou, El Habib Benlahmar

**Affiliations:** 1 Software Project Management Research Team, ENSIAS, Mohammed V University in Rabat, Rabat, Morocco; 2 Laboratory of Information Technology and Modeling, Faculty of Sciences Ben M’sick, Hassan II University, Casablanca, Morocco

**Keywords:** JSON-based databases, Document-oriented data, Schema extraction, Schema evaluation, Schema inference, Schema evolution, Data integration, Benchmarking, Computational biology and bioinformatics, Mathematics and computing

## Abstract

The increasing use of schemaless data systems has intensified the need for reliable methods to assess the quality of extracted schemas intended for downstream tasks such as data integration, query optimisation, and interoperability. Although numerous schema inference techniques have been proposed, the field still lacks standardised and method-independent criteria for evaluating the validity and accuracy of inferred schemas. This paper introduces the Schema Validation and Evaluation Framework (SVEF), a systematic evaluation model for assessing extracted schemas across six complementary dimensions that capture essential structural and semantic properties: Data Type Accuracy, Required and Optional Fields, Multiple Type Support, Collection Structure Consistency, Entity Relationships, and Temporal Evolution Detection. Each dimension is defined through formal, data-driven metrics that quantify the degree to which an inferred schema reflects characteristics observed in the underlying dataset. In the present study, the framework is instantiated and evaluated for schemaless document-oriented data represented in JSON or JSON-like form. SVEF is evaluated using controlled benchmark datasets with curated ground-truth schemas and is applied to three representative schema extraction approaches. The results show that, while existing methods achieve strong performance in basic type reconstruction, substantial differences remain in modelling conditional fields, complex collection structures, and schema evolution over time. SVEF provides a consistent and interpretable basis for comparing schema extraction strategies and supports more rigorous empirical analysis of their behaviour in dynamic document-oriented data environments.

## Introduction

Reliable evaluation of extracted schemas is essential when such schemas are intended for downstream tasks such as data integration, query optimisation, interoperability, and data validation. An extracted schema is useful only to the extent that it reflects the observed data with sufficient accuracy, structural consistency, and practical reliability. This requirement highlights the need for a systematic and reliable approach to evaluating extracted schemas prior to their use. The issue becomes particularly important in schemaless environments, where data instances may vary in structure, evolve over time, and remain only partially documented.

The growing adoption of document-oriented database systems has enabled organisations to manage large volumes of heterogeneous data represented in JSON or JSON-like form. Unlike relational systems, these environments commonly permit structural variability across records, do not require predefined schemas, and allow data structures to evolve without coordinated versioning. Although these characteristics provide flexibility in data management, they also introduce difficulties for integration, querying, interoperability, and systematic understanding of the underlying data. Within this context, automated schema extraction has emerged as an important mechanism for recovering the implicit logical structure of schemaless datasets and supporting higher-level data management tasks^1–3^.

A broad range of schema inference techniques has been proposed, including clustering-based methods, statistical and frequency-driven summarisation, embedding-based similarity models, and other structure-aware approaches. Yet, despite this progress, the literature continues to lack standardised and method-independent criteria for evaluating the quality of extracted schemas. Many existing studies rely on ad hoc indicators, qualitative inspection, dataset-specific assumptions, or partial measurements that address only selected aspects of schema quality. This situation complicates comparison between approaches, limits the interpretability of empirical results, and leaves unclear the extent to which inferred schemas capture semantic dependencies and temporal variation^2,4,5^.

To address this limitation, the paper introduces the Schema Validation and Evaluation Framework, hereafter denoted SVEF. The framework establishes a systematic basis for assessing extracted schemas across six complementary dimensions: Data Type Accuracy, Required and Optional Fields, Multiple Type Support, Collection Structure Consistency, Entity Relationships, and Temporal Evolution Detection. These dimensions reflect key characteristics commonly encountered in real-world schemaless document-oriented data. Each is operationalised through quantitative, data-driven metrics that support consistent and reproducible evaluation across datasets and schema inference approaches.

The broader aim of this work is to contribute to the general problem of extracted-schema evaluation. In the present study, SVEF is designed and evaluated for schemaless document-oriented data represented in JSON or JSON-like form. This scope provides an appropriate basis for treating the evaluation problem with methodological precision and empirical clarity.

By establishing a structured basis for schema evaluation, SVEF extends the analysis of schema extraction methods beyond surface-level structural coverage. It supports systematic comparison between approaches, enables transparent reporting of strengths and limitations, and provides a more rigorous basis for empirical investigation of schema extraction behaviour. Through controlled experiments using benchmark datasets with curated ground-truth schemas, the paper demonstrates that the framework can reveal meaningful differences between methods that remain insufficiently visible under conventional evaluation practices. In this way, the study contributes to a more robust empirical foundation for research on schema extraction in dynamic document-oriented data environments.

The remainder of this article is organised as follows. Section Related work reviews existing approaches to schema extraction and identifies the methodological gap motivating a unified evaluation framework. Section Methodology presents SVEF, including its six validation dimensions and formal metric definitions. Section Experimental evaluation describes the experimental design, datasets, baseline methods, and evaluation setup. Section Discussion discusses the empirical findings, interprets method performance across evaluation dimensions, and outlines limitations and directions for future work. Section Conclusions concludes the paper by summarising the main contributions and reflecting on the importance of more systematic evaluation practices for extracted schemas.

## Related work

Research on schema extraction spans a broad range of methodological perspectives, including structural summarisation, statistical inference, semantic modelling, unified metamodels, and schema evolution. Although the present study is instantiated and evaluated for JSON-based databases, the broader literature also includes adjacent work in XML schema inference and RDF shape extraction. Taken together, these strands have substantially advanced the recovery of implicit structure from heterogeneous and schemaless data sources, yet their evaluation practices remain fragmented and often tailored to specific techniques, formalisms, or datasets. This section organises prior contributions into four major research strands that reflect dominant methodological trends while also clarifying the broader context in which the proposed evaluation framework is positioned.

### Structural and model-driven schema inference

Structural and model-driven techniques represent some of the earliest and most extensively studied approaches to schema extraction in semi-structured and schemaless settings. In the JSON context, Baazizi et al. propose a parametric schema inference algorithm for large-scale datasets based on parameterised types, explicit modelling of unions and arrays, and statistical aggregation of heterogeneous document structures^6^. Their evaluation primarily emphasises scalability and schema reduction, offering limited insight into the accuracy of individual schema components.

Koupil et al. extend this line of work through a universal inference framework supporting relational, document, key–value, columnar and graph databases via a shared representation and systematic mapping rules^7^. While the approach handles structural heterogeneity and schema variants, its evaluation remains centred on global correctness and performance rather than on finer-grained properties such as primitive type precision, union resolution or nested collection regularity.

Related work on structural inference also extends beyond the JSON setting. In the XML domain, Klempa et al. present jInfer, a framework for XML schema inference that supports the implementation, testing, and comparison of inference modules^8^. While not directly comparable to JSON schema extraction pipelines, it reflects a broader view of schema inference as a modular methodological problem across data models. Similarly, studies such as the type inference and validation approach of You et al. address heterogeneous data environments, but their evaluation remains centred on type-level correctness rather than broader structural or temporal behaviour^9^.

A more directly relevant contribution is the comparative study by Latták and Koupil, which examines five JSON schema inference approaches through static and dynamic analysis^10^. The study identifies functional differences, performance trade-offs, and unresolved issues in JSON schema inference, thereby highlighting the methodological difficulty of comparing existing approaches. However, its primary focus is the comparison of inference algorithms, not the definition of an evaluation framework that integrates structural, semantic, and temporal dimensions.

Overall, this strand emphasises structural abstraction, generality, and algorithmic design, but provides limited mechanisms for systematically analysing the fidelity of extracted schemas across multiple quality dimensions.

### Semantic and relationship-aware schema extraction

Semantic and relationship-aware approaches seek to complement structural inference by identifying contextual correspondences between schema elements, latent entity boundaries, referential attributes, and dependency patterns that are not recoverable from surface structure alone. In this context, the term semantic is used in a restricted sense. It refers primarily to context-aware similarity, naming regularities, and relation discovery within the observed data, rather than to ontology grounding or external conceptual validation against a domain knowledge base. In adjacent literatures on semantic data integration, schema matching, and ontology integration, semantic alignment, mapping, and grounding are treated as related but distinct problems, often involving explicit correspondences between heterogeneous schemas or ontologies rather than the internal evaluation of an extracted schema artefact.

Souibgui et al. propose an embedding-based method that augments a global structural schema with graph-derived representations to detect identifiers and references in JSON document stores ^11^. Similarly, Belefqih et al. introduce an SBERT-based schema extraction approach that models JSON documents as typed triplets encoding entities, attributes, and structural links, followed by aggregation into a unified schema^12,13^. These approaches improve relationship discovery and contextual coherence, particularly where structural regularity alone is insufficient to recover the intended organisation of the data.

A related but model-specific line of work appears in the RDF community. Fernández-Álvarez et al. present sheXer, a system that extracts shapes from existing RDF graphs and generates ShEx and SHACL representations. This work addresses the derivation of structural constraints from graph-based semantic data^14^.

Despite the advances made by relationship-aware and context-aware approaches, their evaluations typically remain task-specific, often relying on precision and recall for identifier or reference detection. As a result, they provide limited insight into relationship completeness, dependency fidelity, or the consistency of inferred associations across the broader extracted schema. In the present study, such aspects are evaluated only insofar as they are reflected in extracted-schema artefacts, particularly through dependency-related regularities and entity relationship recovery, rather than through ontology alignment or external semantic grounding.

### Unified metamodels and multimodel representations

Unified metamodels address the heterogeneity of data systems by providing common logical representations that span multiple storage paradigms. Candel et al. introduce U-Schema, a metamodel that formalises entities, attributes, references and structural variations across both NoSQL and relational databases^15^. This abstraction enables consistent representation of diverse schema elements and serves as a foundation for schema inference and transformation tools. Related multimodel representation frameworks proposed by Chillon et al. and related work map document, relational, key–value, columnar and graph data into shared intermediate structures, thereby facilitating systematic comparison across storage paradigms^7,16^. Broader surveys of NoSQL data modelling further emphasise the importance of such abstractions, highlighting the diversity of logical models and the absence of convergent representation standards^17,18^.

Although these contributions provide strong modelling foundations, their emphasis lies primarily on representation design and transformation rules. They generally do not offer systematic measures for assessing how accurately inferred schemas populate these representations or how faithfully individual schema elements reflect the underlying data.

### Schema evolution and temporal modelling

Schema evolution and temporal modelling constitute a long-standing research direction in schemaless data management. Early work such as the τJSchema framework by Brahmia et al. demonstrates how timestamped JSON schema versions can be represented and queried to support temporal reasoning over evolving datasets^19,20^. This research highlights the importance of modelling schema changes explicitly rather than treating schemas as static artefacts.

Subsequent studies extend this foundation by proposing generic evolution mechanisms and structured change taxonomies. Chillon et al. introduce a schema evolution approach applicable to both NoSQL and relational systems, including the Orion evolution language and a catalogue of schema operations that can be applied consistently across data models^16^. Fedushko et al. focus on safe migration strategies for document databases, addressing correctness and service continuity during schema transitions^21^.

In the context of graph databases, Hausler and Klettke present Nautilus, an implementation of an evolution approach that supports schema differencing, change detection and structural transformation for graph-oriented data^22^. Their work demonstrates how evolution primitives and version management can be operationalised in practice, particularly in environments where schema changes are implicit or decentralised.

Comprehensive surveys, such as the review by Brahmia et al., synthesise these contributions across relational, XML and NoSQL systems and consistently identify the absence of standard metrics for evaluating temporal correctness and evolution fidelity^23^.

### Positioning of proposed evaluation framework

Taken together, the literature reviewed above reflects substantial progress in schema inference, semantic enrichment, unified modelling, and schema evolution. However, evaluation practices across these strands remain largely isolated and technique-specific. In this context, structural aspects are evaluated directly across all dimensions, whereas semantic aspects are treated more narrowly through dependency-related regularities and relationship recovery, rather than through ontology-level alignment or grounding. As summarised in Table 1, most studies assess selected properties in isolation, such as type accuracy, relationship precision, or migration correctness, without a coherent framework that integrates structural, semantic, and temporal aspects.

**Table 1:** Summary of major research strands in schema extraction and their evaluation gaps.

Research strand	Representative methods	Primary capabilities	Evaluation limitations
Structural and model-driven schema inference	Parametric Inference (Baazizi et al.) ; Universal Inference (Koupil et al.) ; U-Schema	Captures structural variety, unions, arrays and nested objects; scalable summarisation of large JSON datasets	Focus on reduction quality and global correctness; limited analysis of primitive types, unions, array regularity, or conditional behaviours
Semantic and relationship-aware extraction	Embedding-based Extraction (Souibgui et al.); BERT-based / SBERT-RDF Extraction; (Belefqih et al.)	Detects identifiers, references, entity boundaries and semantic links using embeddings or linguistic cues	Evaluations rely on task-specific precision and recall; limited insight into dependency fidelity, relationship completeness, or spurious link generation
Unified metamodels and multimodel representations	U-Schema (Candel et al.) ; Multimodel Mapping (Chillon et al., Koupil et al.)	Provides uniform logical representation across document, relational, graph and key–value models	Strong modelling focus, but no systematic measures for the accuracy of inferred schema population or for type and array fidelity
Schema evolution and temporal modelling	τJSchema (Temporal et al.) ; Orion (Chillon et al., Koupil et al.) Nautilus (Hausler and Klettke)	Models schema versions, change operations, migrations, and temporal dependencies; supports snapshot comparison and evolution management	Lack of standard metrics for assessing temporal correctness and fidelity of inferred evolution patterns; evaluations often focus on migration correctness or runtime
Surveys and meta-analyses	SLR (Belefqih et al.); Temporal Survey (Brahmia et al.)	Document methodological diversity and fragmented evaluation practices	Identify gaps but do not provide metric-based evaluation frameworks
Adjacent schema extraction in XML and RDF	jInfer (Klempa et al.); sheXer (Fernández-Álvarez)	XML schema inference; RDF shape extraction in ShEx/SHACL; support for modular inference and structure derivation	Model-specific formalisms; not directly comparable to JSON extraction metrics; limited support for unified cross-model quality assessment

The evaluation framework introduced in this work is positioned as a response to this gap. Rather than proposing a new extraction method, it provides a systematic, metric-based model for assessing inferred schemas across complementary quality dimensions. This positioning allows existing and future inference approaches to be compared on a common basis, supporting more transparent analysis and facilitating deeper understanding of trade-offs between schema properties.

## Methodology

**Fig. 1:**
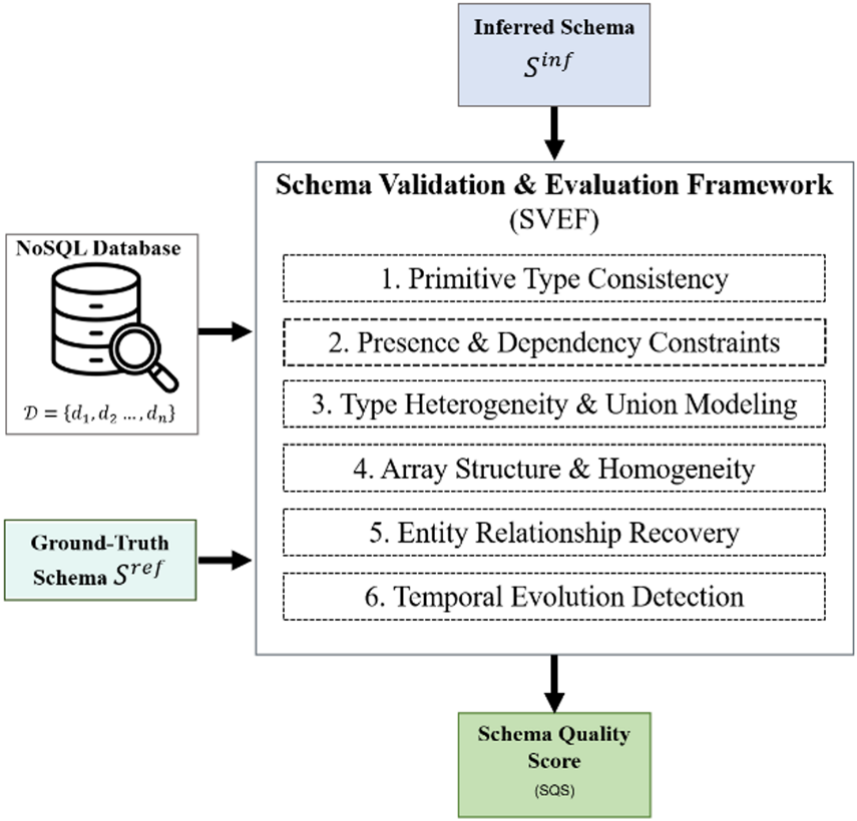
Overview of the SVEF.

The Schema Validation and Evaluation Framework, abbreviated as SVEF, provides the methodological basis of this study. As shown in Fig. 1, it defines a structured process for assessing the quality of schemas inferred from schemaless document-oriented databases represented in JSON or JSON-like form. The framework is intended to examine how accurately an inferred schema reflects the structural organisation, semantic dependencies, and temporal behaviour observed in the underlying dataset.

SVEF organises schema evaluation into six analytical dimensions, each addressing a distinct aspect of schema quality. Together, these dimensions cover core properties of schema validity, including type consistency, property presence and dependency patterns, heterogeneous typing, array organisation, inter-entity relationships, and schema evolution over time. This structure supports both fine-grained analysis and aggregated evaluation, and provides a consistent basis for empirical validation and comparative assessment.

In the context of this paper, the term schema denotes a structured representation of the organisation of a JSON-based dataset. It is not restricted to a standard JSON Schema document, but refers more generally to the representation of properties, types, required and optional fields, array structures, inter-entity relationships, and temporal variants when relevant. Accordingly, both inferred and reference schemas are treated through this common abstract representation for the purposes of evaluation within SVEF.

### Framework overview

The framework takes as input a dataset, denoted by $\mathcal{D}=\left\{{d}_{1},{d}_{2}\dots ,{d}_{n}\right\}$, composed of schemaless records represented in JSON or JSON-like form. From this dataset, a schema inference process generates an inferred schema ${S}^{inf}$, which captures the structural and semantic organisation identified by the extraction method. The quality of this inferred schema is then evaluated either against a reference schema ${S}^{ref}$, when a ground-truth or expert-defined schema is available, or against structural evidence derived directly from the dataset.

The evaluation proceeds through six analytical stages corresponding to the validation dimensions introduced above. Each stage addresses a distinct aspect of schema quality, ranging from the consistency of atomic types to the detection of temporal evolution patterns. For each dimension, SVEF applies a set of quantitative indicators ${M}_{i}$ that measure the correspondence between the inferred and the reference representations. These indicators are then combined into a dimension-level score ${S}_{i}$ , which expresses the degree of conformance for the aspect under consideration.

To clarify the interpretation of these dimensions, the framework is illustrated using a compact ecommerce example. Consider a collection of order documents containing properties such as *order_id*, *customer_id*, *items*, *payment_method*, *discount_code*, and *shipping_address*. Some properties are expected to occur in all records, whereas others appear only under specific conditions. For instance, *discount_code* may be present only in discounted transactions, *payment_details* may vary according to the payment method, and *items* may consist of an array of nested product objects. Across temporal snapshots, new properties such as *delivery_status* may be introduced, and existing structures such as payment information may evolve from a primitive value to a nested object. This example is used throughout the following subsections to illustrate how each validation dimension captures a particular aspect of extracted-schema quality.

The global evaluation result is obtained by aggregating the six dimension-level scores into an overall Schema Quality Score $SQS$, computed as a weighted combination:

1 $$SQS = \mathop \sum \limits_{i = 1}^{6} w_{i} \times S_{i} , \mathop \sum \limits_{i} w_{i} = 1$$

The weights $w_{i}$ can be adjusted to reflect particular evaluation priorities, such as assigning greater importance to structural precision or to temporal behaviour. Through this formulation, SVEF provides a unified quantitative basis for the consistent and comparable assessment of schema inference approaches across datasets and evaluation contexts.

### Data type accuracy

The first dimension of the proposed framework concerns the correctness of primitive type inference in the extracted schema. In schemaless data environments, a schema should accurately represent the atomic data types observed in the underlying dataset. Although primitive categories such as string, number, and boolean may appear straightforward, their correct identification provides the basis for higher-level structural and semantic interpretation. Inaccurate primitive typing can therefore introduce inconsistencies that propagate to subsequent stages of schema analysis and affect downstream tasks such as validation, interoperability and data integration.

Within the running ecommerce example, this dimension evaluates whether properties such as *order_id*, *price*, or *is_expedited* are assigned the primitive types actually observed in the data. For instance, a robust inferred schema should recognise *order_id* as a string identifier, *price* as a numeric value when appropriate, and *is_expedited* as a boolean attribute, rather than representing such properties through incorrect or overly generic types.

To evaluate this dimension, the framework compares the atomic types observed directly in the dataset with those declared in the inferred schema. For each property $p$, the observed set of data types, denoted $PT_{p}^{obs}$, is derived from empirical inspection of document instances, while $PT_{p}^{inf}$ denotes the set of types assigned to the same property by the inference algorithm. The degree of conformance is quantified through a normalized similarity measure that captures the overlap between these two sets:

2 $$Type\;Conformance\left( p \right) = \frac{{\left| {PT_{p}^{obs} \cap PT_{p}^{\inf } } \right|}}{{\left| {PT_{p}^{obs} \cup PT_{p}^{\inf } } \right|}}$$

This measure yields a continuous score between 0 and 1, where values close to 1 indicate strong agreement between inferred and observed primitive types.

The overall dataset-level score, denoted Data Type Accuracy (DTA), is computed as the arithmetic mean of the property-level conformance values across all properties in the schema:

3 $$DTA = \frac{1}{{\left| {\rm P} \right|}}\mathop \sum \limits_{{p \in {\rm P} }} Type\;Conformance \left( p \right)$$

A high DTA score indicates that the schema inference approach preserves primitive typing information with sufficient accuracy to support coherent structural interpretation. By contrast, lower scores reveal weaknesses in type abstraction, such as representing numeric attributes as strings or collapsing distinct primitive categories into overly generic representations. In this way, the metric provides a direct basis for assessing one of the most fundamental aspects of extracted-schema quality.

### Required and optional fields

A second dimension of schema evaluation concerns the accurate representation of property presence and inter-attribute dependencies. In schemaless environments, records belonging to the same collection may exhibit substantial structural variation, with some properties appearing systematically and others occurring only under specific conditions. Representing this variation is not only a structural matter; it also reflects implicit constraints in the data, including conditional existence patterns and recurrent co-occurrence relations.

In the running ecommerce example, properties such as *order_id* or *customer_id* may be expected in all order documents, whereas *discount_code* may appear only in discounted transactions. Similarly, the presence of *payment_method* may imply the appearance of additional attributes such as *payment_details* or *transaction_fee*. These patterns illustrate the need for an inferred schema to distinguish mandatory properties from optional ones and to capture regular dependencies between fields.

The framework evaluates this dimension by examining the extent to which the inferred schema reproduces the requiredness and dependency relations observed in the dataset. For each property $p$, the empirical frequency of occurrence across all documents, denoted $f_{p}$, serves as an indicator of its presence pattern. A property is considered required when $f_{p}$ exceeds a predefined empirical threshold and optional otherwise.

Let $R_{p}^{inf}$ and $R_{p}^{ref}$ denote, respectively, the requiredness status inferred by the extraction algorithm and that defined in the reference schema or empirical baseline. The proportion of correctly identified presence conditions across all properties is then expressed as

4 $$\Pr esence\;Accuracy = \frac{1}{{\left| {\rm P} \right|}}\mathop \sum \limits_{{p \in {\rm P} }} 1\left[ {R_{p}^{\inf } = R_{p}^{ref} } \right]$$

where the indicator function 1[⋅] equals 1 when the two designations coincide. This measure captures the ability of the inference process to distinguish mandatory attributes from contextually optional ones, thereby avoiding schemas that are either overly restrictive or excessively permissive.

Beyond individual presence conditions, the framework also evaluates dependency constraints, namely logical relations expressing the co-occurrence or conditional appearance of multiple attributes. Such dependencies are derived from the empirical distribution of properties within the dataset and represented as association rules of the form $A \Rightarrow B$. For each candidate rule, the framework computes support, confidence and lift, defined respectively as:

5 $${\mathrm{Supp}}\left( {{\mathrm{A}},{\mathrm{B}}} \right) = {\mathrm{P}}\left( {{\mathrm{A}} \wedge {\mathrm{B}}} \right)$$

6 $${\mathrm{Conf}}\left( {{\mathrm{A}} \Rightarrow {\mathrm{B}}} \right) = \frac{{{\mathrm{Supp}}\left( {{\mathrm{A}},{\mathrm{B}}} \right)}}{{{\mathrm{P}}\left( {\mathrm{A}} \right)}}$$

7 $${\mathrm{Lift}}\left( {{\mathrm{A}} \Rightarrow {\mathrm{B}}} \right) = \frac{{{\mathrm{Conf}}\left( {{\mathrm{A}} \Rightarrow {\mathrm{B}}} \right)}}{{{\mathrm{P}}\left( {\mathrm{B}} \right)}}$$

A dependency is considered successfully recovered when its inferred confidence and lift exceed empirically defined thresholds, commonly $\tau_{conf} = 0.8$ and $\tau_{lift} = 1.2$. The overall quality of dependency recovery can then be assessed through precision and recall over the set of valid reference rules.

Taken together, the presence-based and dependency-based components provide a structured view of how well the inferred schema captures intra-document regularities and cross-attribute constraints. High scores indicate that the schema reflects both stable and conditional property patterns with reasonable accuracy. Lower scores suggest either excessive generalisation, which weakens structural specificity, or overfitting to infrequent combinations of attributes, which reduces the interpretability and reuse value of the extracted schema.

### Multiple type support

A further dimension of schema evaluation concerns the ability of an inferred schema to represent heterogeneous typing patterns that arise naturally in schemaless data collections. Unlike relational systems, where each attribute is associated with a fixed type, document-oriented data may assign different primitive or composite types to the same property across records. For example, a field such as *price* may appear as a numeric value in some instances and as a string containing a currency symbol in others, while a property such as *shipping_address* may be represented either as free text or as a nested object.

In the running ecommerce example, such variation is not necessarily anomalous. Rather, it reflects the flexible and evolving character of schemaless data. A reliable inferred schema should therefore capture legitimate type diversity through appropriate union types or type alternatives, while avoiding unsupported type generalization.

Within the proposed framework, this dimension is evaluated by measuring the correspondence between the set of distinct types empirically observed for each property and the set of types declared in the inferred schema. For a given property $p$ let $T_{p}^{obs}$ denote the set of unique atomic or composite types observed in the dataset and $T_{p}^{inf}$ denote the corresponding set inferred by the schema extraction algorithm. Two complementary aspects are examined: (i) whether the schema successfully captures all observed variants and (ii) whether it introduces superfluous or overly permissive type alternatives that are not supported by the data. These aspects are jointly expressed through a union conformance score, defined as

8 $${\mathrm{MTS}}\left( {\mathrm{p}} \right) = \frac{{\left| {{\mathrm{T}}_{{\mathrm{p}}}^{{{\mathrm{obs}}}} \cap {\mathrm{T}}_{{\mathrm{p}}}^{{{\mathrm{inf}}}} } \right|}}{{\left| {{\mathrm{T}}_{{\mathrm{p}}}^{{{\mathrm{obs}}}} } \right|}} - {\lambda }\frac{{\max \left( {0,\left| {{\mathrm{T}}_{{\mathrm{p}}}^{{{\mathrm{inf}}}} } \right| - \left| {{\mathrm{T}}_{{\mathrm{p}}}^{{{\mathrm{obs}}}} } \right|} \right)}}{{\left| {{\mathrm{T}}_{{\mathrm{p}}}^{{{\mathrm{inf}}}} } \right|}}$$

The first term measures the coverage of empirically observed type variants, whereas the second penalises overgeneralization. The parameter $\lambda$ controls the relative weight of this penalty. The overall score for this dimension is obtained by averaging $MTS\left( p \right)$ over properties in the schema.

High scores indicate that the inferred schema captures legitimate type variation while remaining bounded by empirical evidence. Lower scores suggest either under-representation of true type diversity or the introduction of unsupported alternatives that weaken the precision of the schema. In this way, the metric evaluates both the flexibility and the discipline of the inference process, both of which are necessary for a reliable representation of heterogeneous schemaless data.

### Collection structure consistency

Another dimension of schema evaluation concerns the structural characterisation of arrays and the homogeneity of their constituent elements. In document-oriented data, arrays are among the most flexible structural constructs. They may represent homogeneous lists of atomic values, collections of nested objects, embedded arrays, or more irregular combinations of elements. Within SVEF, arrays, objects, and primitive values are treated as distinct type categories. An array–object discrepancy is therefore counted as a type mismatch, while the internal structure of correctly identified arrays is assessed separately through the Collection Structure Consistency dimension.

In the running ecommerce example, the field *items* may consist of an array of product objects, each containing properties such as *product_id*, *quantity*, and *price*. A reliable inferred schema should therefore recover both the fact that *items* is an array and the internal regularity of its elements. If the array is flattened, treated as an unconstrained container, or assigned an incorrect nesting level, the resulting schema will provide only a partial representation of the data structure.

Within the proposed framework, the analysis of array structures focuses on two complementary aspects: (i) the structural depth and nesting regularity of arrays and (ii) the type homogeneity of their contained items. The first aspect captures how accurately the inference method reconstructs the hierarchical levels of embedded arrays, whereas the second examines whether the elements within a given array follow a stable type pattern or exhibit uncontrolled heterogeneity.

For each array property $a$, the empirical structure observed in the dataset is characterized by its typical nesting depth $d_{a}^{obs}$ and by the set of item types $I_{a}^{obs}$. The corresponding inferred schema specifies its own estimates $d_{a}^{inf}$ and $I_{a}^{inf}$. The degree of structural alignment is assessed through a depth conformance score:

9 $${\mathrm{DepthConformance}}\left( {\mathrm{a}} \right) = 1 - { }\frac{{\left| {{\mathrm{d}}_{{\mathrm{a}}}^{{{\mathrm{obs}}}} { } - {\text{ d}}_{{\mathrm{d}}}^{{{\mathrm{inf}}}} } \right|}}{{1 + \left| {{\mathrm{d}}_{{\mathrm{a}}}^{{{\mathrm{obs}}}} { } - {\text{ d}}_{{\mathrm{d}}}^{{{\mathrm{inf}}}} } \right|}}$$

This score approaches 1 when the inferred and observed nesting levels coincide. To evaluate internal uniformity, the framework computes a homogeneity index based on the entropy of the type distribution among array items:

10 $${\mathrm{Homogeneity}}\left( {\mathrm{a}} \right) = 1 - { }\frac{{{\mathrm{H}}\left( {{\mathrm{I}}_{{\mathrm{a}}}^{{{\mathrm{obs}}}} } \right)}}{{{\mathrm{log}}\left( {\left| {{\mathrm{I}}_{{\mathrm{a}}}^{{{\mathrm{obs}}}} } \right| + 1} \right)}}$$

where $H\left( . \right)$ denotes Shannon entropy. A value close to 1 indicates that the array predominantly contains elements of a single type, whereas lower values reflect increasing type diversity within the same array. The overall Collection Structure Consistency (CSC) score combines these two components through a weighted aggregation:

11 $$CSC = \frac{1}{\left| A \right|} \sum\limits_{a \in A } {\left( {\alpha \cdot Homogeneity\left( a \right) + \left( {1 - \alpha } \right) \cdot DepthConformance\left( a \right)} \right)}$$

where $\alpha$ represents the relative importance assigned to type uniformity versus structural depth.

This dimension provides a quantitative basis for assessing how faithfully the inferred schema captures both the composition and the complexity of array structures. High $CSC$ scores indicate that the inference process preserves array organisation without flattening nested structures or introducing unnecessary heterogeneity. Lower scores suggest that the inferred schema fails to reproduce important regularities in array depth or internal composition, thereby reducing its value for downstream validation, transformation, and integration tasks.

### Entity relationships recovery

A further dimension of schema evaluation concerns the ability of the inferred schema to recover and formalise inter-entity relationships. In document-oriented databases, such relationships are often expressed implicitly through nested objects, repeated references, or shared identifiers rather than through explicit foreign keys. Recovering these relations is important for preserving the semantic organisation of the data model and for supporting downstream tasks such as integration, querying, and semantic interpretation.

In the running ecommerce example, relationships may appear in two main forms. The first is aggregation, where an *order* document contains an *items* array whose elements correspond to product-related entities embedded within the order structure. The second is reference, where a field such as c*ustomer_id* links an order to a customer entity without requiring an explicit relational constraint. A reliable inferred schema should therefore capture both containment-based and reference-based relations where they are supported by the data.

Within the proposed framework, each inferred schema is represented as a directed labeled graph $G^{inf} = \left( {V^{inf} ,E^{inf} } \right)$, where vertices $V$ denote entities or document types and edges $E$ encode the relationships among them. An analogous graph $G^{ref}$ is derived from the reference schema or from a manually annotated gold standard.

Evaluating relationship recovery thus becomes a graph-alignment problem in which edges are compared according to their source and target entities, their relationship type, and their multiplicity. Let $TP$, $FP$ and $FN$ denote, respectively, the number of correctly identified, spurious and missed relationships. Classical information-retrieval metrics are then used to quantify edge-level performance:

12 $${\mathrm{Precision}} = \frac{{{\mathrm{TP}}}}{{{\mathrm{TP}} + {\mathrm{FP}}}}$$

13 $${\mathrm{Recall}} = \frac{{{\mathrm{TP}}}}{{{\mathrm{TP}} + {\mathrm{FN}}}}$$

14 $${\mathrm{F}}_{1} = 2{ } \times \frac{{{\mathrm{Precision}} \times {\mathrm{Recall}}}}{{{\mathrm{Precision}} + {\mathrm{Recall}}}}$$

To complement these local indicators, SVEF also incorporates a structural similarity measure based on the normalized graph edit distance between the inferred and reference relationship graphs:

15 $${\mathrm{GED}}_{{{\mathrm{norm}}}} = 1 - \frac{{{\mathrm{GED}}\left( {{\mathrm{G}}^{{{\mathrm{inf}}}} ,{\mathrm{G}}^{{{\mathrm{ref}}}} } \right)}}{{\left| {{\mathrm{E}}^{{{\mathrm{ref}}}} } \right|}}$$

where $GED\left( . \right)$ denotes the minimal number of edge insertions, deletions, or relabelings required to transform one graph into the other. The overall Entity relationship recovery score, denoted ERR, is then computed as a weighted combination of local and global indicators:

16 $$ERR = \beta \cdot F_{1} + \left( {1 - \beta } \right) \cdot GED_{norm}$$

where $\beta$ empirically set to 0.7 in order to balance edge-level correctness and global structural similarity.

High ERR scores indicate that the inferred schema reconstructs both direct and nested inter-entity dependencies with sufficient accuracy to preserve the semantic organisation of the dataset. Lower scores suggest missed links, incorrect associations, or structural distortions in the inferred relationship graph. By combining precision–recall measures with global graph similarity, this dimension evaluates not only the correctness of individual relations but also the coherence of the relational structure captured by the schema.

### Temporal evolution detection

The final dimension of the proposed framework examines the ability of a schema inference approach to identify and characterise temporal evolution in schemaless datasets. In document-oriented environments, data structures do not remain fixed. New attributes may be introduced, existing ones may disappear, and previously simple properties may evolve into more complex nested forms. Since such changes are often reflected only in successive states of the stored documents, schema evaluation must consider not only structural correctness at a given point in time, but also the capacity to capture structural variation across time.

In the running ecommerce example, temporal evolution may appear through changes such as the introduction of a new property *delivery_status*, the removal of a deprecated attribute, or the transformation of payment information from a primitive field into a nested object. These changes illustrate that an inferred schema should not only represent the current state of the data, but also reflect how the structure evolves across temporal snapshots.

Within the SVEF framework, the evaluation of schema evolution focuses on two complementary aspects: (i) the detection of distinct schema versions, corresponding to structural configurations observed over time; and (ii) the accuracy with which transitions between these versions are identified. Let the dataset be partitioned into a chronological sequence of temporal windows ${ mathcal{D}} = \left\{ {{ mathcal{D}}_{{t_{1} }} , \ldots ,{ mathcal{D}}_{{t_{T} }} } \right\}$, where each window represents a bounded interval or snapshot. For each period $t_{i}$ , the inference process yields a schema $S_{{t_{i} }}^{inf}$, which is compared with a reference schema $S_{{t_{i} }}^{ref}$ or with synthetic ground truth when such a reference is available.

The Version Detection Accuracy, denoted $VDA$, measures the proportion of temporal windows for which the inferred schema correctly identifies the structural variant present at that time:

17 $${\mathrm{VDA}} = \frac{{\left| {\left\{ {{\mathrm{t}}_{{\mathrm{i}}} { }:{\text{ S}}_{{{\mathrm{t}}_{{\mathrm{i}}} }}^{{{\mathrm{inf}}}} \approx {\mathrm{S}}_{{{\mathrm{t}}_{{\mathrm{i}}} }}^{{{\mathrm{ref}}}} } \right\}} \right|}}{{\mathrm{T}}}$$

where the symbol $\approx$ denotes structural equivalence based on element-level matching. Complementarily, the inferred Change Detection Rate, denoted $CDR^{inf}$, measures the responsiveness of the schema inference process to structural drift across consecutive time windows:

18 $${\mathrm{CDR}}^{{{\mathrm{inf}}}} = \frac{{\left| {\left\{ {{\mathrm{t}}_{{\mathrm{i}}} { }:{\text{ S}}_{{{\mathrm{t}}_{{\mathrm{i}}} }}^{{{\mathrm{inf}}}} \ne {\mathrm{S}}_{{{\mathrm{t}}_{{{\mathrm{i}} - 1}} }}^{{{\mathrm{inf}}}} } \right\}} \right|}}{{{\mathrm{T}} - 1}}$$

The reference rate of structural evolution, denoted $CDR^{ref}$, is defined analogously over the sequence of reference schemas:

19 $${\mathrm{CDR}}^{{{\mathrm{ref}}}} = \frac{{\left| {\left\{ {{\mathrm{t}}_{{\mathrm{i}}} { }:{\text{ S}}_{{{\mathrm{t}}_{{\mathrm{i}}} }}^{{{\mathrm{ref}}}} \ne {\mathrm{S}}_{{{\mathrm{t}}_{{{\mathrm{i}} - 1}} }}^{{{\mathrm{ref}}}} } \right\}} \right|}}{{{\mathrm{T}} - 1}}$$

Comparing $CDR^{inf}$ with $CDR^{ref}$ allows the framework to assess both over-sensitivity, corresponding to spurious version detection, and under-sensitivity, corresponding to missed changes. The overall Temporal Evolution Detection score, denoted $TED$, combines these two aspects as follows:

20 $$TED = \frac{1}{2} \left( {VDA + \left[ {1 - \left| {CDR^{\inf } - CDR^{ref} } \right|} \right]} \right)$$

This score takes values between 0 and 1, where higher values indicate stronger agreement between inferred and observed evolution patterns.

This dimension extends schema evaluation from a static perspective to a temporal one. High TED scores indicate that the inference process captures both structural continuity and genuine points of change with reasonable accuracy. Lower scores suggest either insufficient sensitivity to gradual schema drift or excessive instability that produces unjustified fragmentation into multiple schema versions. In this way, temporal evolution detection contributes to a more complete assessment of extracted-schema quality by accounting for the continuity and adaptability of schema representations across time. For clarity, Table 2 summarises the principal notation used in SVEF.

**Table 2:** Principal notation used in SVEF.

Symbol/Abbreviation	Meaning
$\mathcal{D}=\left\{{d}_{1},{d}_{2}\dots ,{d}_{n}\right\}$	Input dataset
${S}^{inf}$	Inferred schema
${S}^{ref}$	Reference schema
${S}_{i}$	Score of validation dimension
${w}_{i}$	Weight of validation dimension in the global score
$SQS$	Overall schema quality score
${PT}_{p}^{obs}$, ${PT}_{p}^{inf}$	Observed and inferred primitive types for property
$DTA$	Data type accuracy
${T}_{p}^{obs}$, ${T}_{p}^{inf}$	Observed and inferred types for property, including primitive and composite forms
$MTS$	Multiple type support
$CSC$	Collection structure consistency
${G}^{inf},{G}^{ref}$	Inferred and reference relationship graphs
$ERR$	Entity relationship recovery
$VDA$	Version detection accuracy
${CDR}^{inf}$,${CDR}^{ref}$	Inferred and reference change detection rates
$TED$	Temporal evolution detection

## Experimental evaluation

This section presents the empirical assessment of the proposed Schema Validation and Evaluation Framework (SVEF). The evaluation examines how effectively SVEF measures the correctness and completeness of inferred schemas across multiple dimensions of schema quality, and how it supports comparison between representative schema extraction approaches. The study is conducted on three heterogeneous benchmark datasets designed to expose different forms of structural variability commonly encountered in schemaless document-oriented data represented in JSON or JSON-like form. All experiments were performed under identical conditions using curated reference schemas and controlled inference outputs.

### Datasets

The evaluation is based on three synthetic yet structurally realistic datasets—E-Commerce, Healthcare and IoT—each selected to stress a distinct subset of schema characteristics relevant to SVEF’s validation dimensions. Collectively, they span a broad spectrum of NoSQL variability: heterogeneous document structures, optional and conditionally dependent fields, union-typed attributes, nested arrays, inter-entity references and temporal schema shifts. This diversity ensures that the six dimensions of SVEF are exercised under controlled but realistically complex conditions. A summary of the dataset characteristics is provided in Table 3.

**Table 3:** Summary of dataset characteristics.

Dataset name	IoT	E-Commerce	Healthcare
Structural complexity	Medium	High	Very High
Type heterogeneity	Low	Medium	High
Optional/dependent properties	Limited optionality (battery_level)	Yes (e.g., age, premium) with dependency chains	Extensive optionality and conditionality (e.g., allergies → medical_history)
Array structures	Structured 1-level arrays for sensor readings	Minimal	Deep nested Medical and Medication arrays
Entity relationships	None	1:N (user → order)	1:N (patient → treatment)
Temporal evolution patterns	Single property addition (battery_level)	Property addition (loyalty_points), type migration (total_amount)	Property addition, removal and multi-phase type evolution (dosage)

The three datasets expose complementary forms of structural and semantic complexity. The E-Commerce dataset combines medium heterogeneity with clear optionality rules, simple foreign-key references (user–order) and two controlled schema evolution events: the mid-timeline addition of loyalty_points and a late-phase type migration of total_amount. The Healthcare dataset introduces the highest structural variability, including deeply nested arrays (medical histories, medication lists), multi-variant union types (for dosage and emergency_contact) and multiple evolution events such as property additions, removals and type shifts across three temporal versions. The IoT dataset provides a more regular structure dominated by nested arrays and a lightweight evolution pattern involving the late introduction of battery_level. Together, these datasets allow systematic assessment across requiredness patterns, type diversity, array behavior, entity relationships and temporal drift.

For each dataset, a curated ground-truth schema was defined to encode the intended structural and semantic properties of the domain. These schemas specify the expected entity structures, required and optional fields, conditional dependencies, admissible type variants, array configurations, inter-entity references and the precise points at which schema evolution occurs. Because the datasets are generated directly from these reference specifications, the resulting ground truth offers a complete and unambiguous baseline against which the correctness of inferred schemas can be measured, enabling a controlled evaluation of each SVEF dimension without interference from real-world noise or undocumented variability.

### Baseline approaches

To contextualise the performance of SVEF, three schema extraction approaches were selected as baselines. The purpose of this selection is not to cover the full range of existing methods, but to represent distinct methodological families within schema inference for JSON-based and document-oriented data. In this sense, the comparison was designed to include approaches that differ in how they model structural regularity, semantic enrichment, and schema evolution, while remaining relevant to the scope of the present study.

The *SBERT-RDF* approach represents a semantic-oriented baseline in which schema elements are expressed as RDF classes and properties enriched through sentence-level embeddings. This method captures primitive datatypes, basic structural relations, and multiple type variants, but does not explicitly model presence constraints or temporal changes beyond snapshot-level extraction^13^.

The *Generic Unified Schema + Orion* configuration represents a structurally oriented baseline that integrates document-level variability through structural variations and aggregate types. It supports explicit datatypes, nested structures, and relationship extraction, while offering more limited support for dependency constraints and array-level homogeneity analysis. Evolution is handled through explicit versioned transformations defined between successive schema states^16^.

The *GEO + Nautilus* pipeline represents a graph-differencing baseline in which schema structure is encoded as a structural graph and evolution is captured through graph-edit operations. This approach supports direct modelling of entity relationships and structural drift, although arrays are not represented explicitly and union types are only indirectly reflected through property-level variations^22^.

Taken together, these baselines provide a comparison space that spans three complementary perspectives on schema inference: semantic representation, structural modelling, and evolution-oriented differencing. This selection was intended to provide methodological diversity within the document-oriented scope of the study, rather than an exhaustive coverage of all available approaches.

### Experimental setup

All experiments were conducted using a unified evaluation pipeline that applies SVEF’s six validation dimensions to each inferred schema across all datasets and baseline methods. For every dataset–method pair, the evaluator processes the curated ground-truth schema, the inferred schema produced by the baseline approach and the full document collection. The evaluation computation follows directly from the formal metric definitions introduced in the methodology section, ensuring consistency between the theoretical framework and its empirical implementation.

Parameter settings governing presence thresholds, type-heterogeneity penalties, array entropy normalization, relationship similarity weighting and temporal-evolution alignment were held constant across all experiments. This configuration is summarized in Table 4, which reports all evaluator parameters extracted directly from the implementation. The complete evaluation logic is outlined in Algorithm 1, providing a concise pseudo-code representation of the unified SVEF procedure. Using fixed parameters ensures that the resulting measurements reflect genuine differences in schema inference quality rather than variations in evaluator configuration.

**Table 4:** Experimental parameters used in SVEF evaluation.

Category	Parameter	Value	Description
Global evaluation	Dimension weights	DTA: 0.20, ROF: 0.15, MTS: 0.15, CSC: 0.15, ERR: 0.20, TED: 0.15	Weighting coefficients for computing the aggregated Schema Quality Score (SQS) (Eq. 1)
Required and optional fields	Presence threshold	0.90	Minimum empirical frequency required for a property to be considered “required” in empirical presence estimation
	Dependency matching rule	Exact set equality	Logical dependencies are considered correct only when inferred antecedent sets match ground truth exactly
Multiple type support	Penalty coefficient λ	0.5	Controls penalty for over-generalized type unions relative to observed coverage (Eq. 8)
Collection Structure Consistency	Homogeneity–depth trade-off α	0.7	Balances type homogeneity (entropy-based) and nesting-depth conformance in the CSC score (Eq. 11)
Entity relationships recovery	Relationship matching rule	Normalized (source, target) pairs	Relationships are matched on normalized entity names, ignoring non-critical attribute differences
	ERR balancing coefficient β	0.7	Weighs F1-score against graph-edit-distance normalization in ERR score (Eq. 16)
Temporal evolution detection	Reference CDR	1.0	Ideal change detection rate against which inferred detection is compared

Each inferred schema was processed independently, but within an identical execution environment to ensure comparability. For every method, SVEF computes its dimension-specific scores—Data Type Accuracy, Required and Optional Fields, Multiple Type Support, Collection Structure Consistency, Entity Relationships and Temporal Evolution Detection—followed by the aggregated Schema Quality Score. The result is a comprehensive, multi-perspective evaluation that allows both fine-grained analysis and summary-level comparison across inference strategies.

**Algorithm 1:**
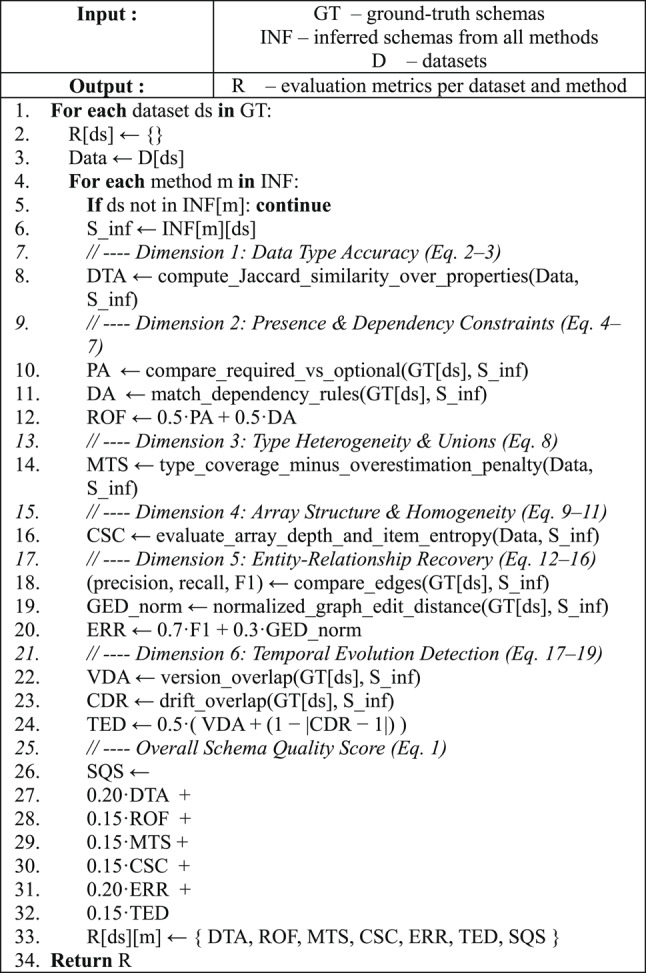
Validation and Evaluation Framework.

## Results

**Fig. 2:**
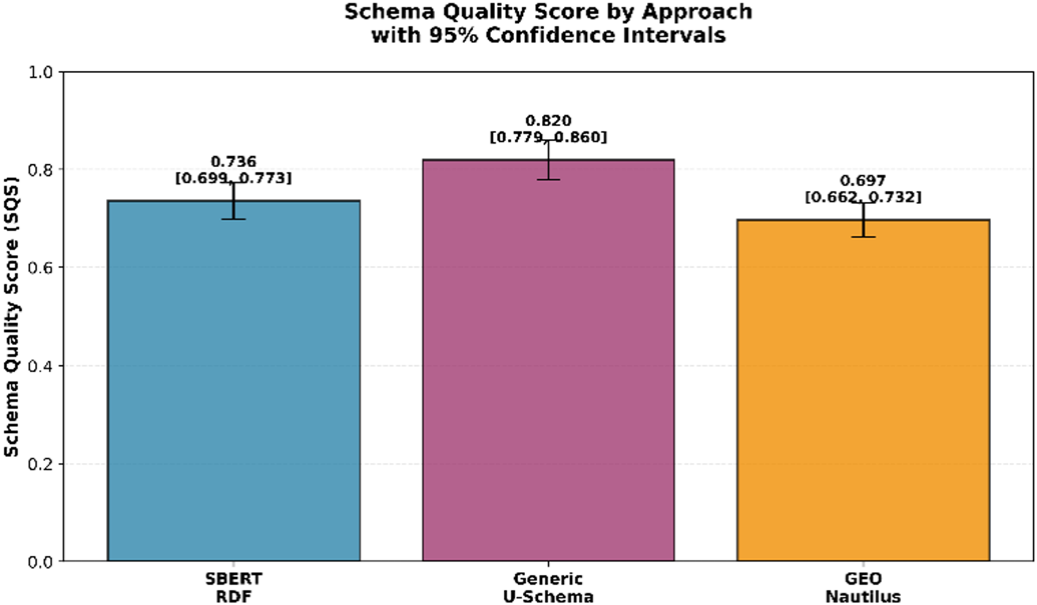
Schema quality score by approach with 95% confidence intervals.

The evaluation results reveal clear performance differences between the three schema inference approaches when assessed under SVEF’s multi-dimensional framework. At the aggregate level, Generic U-Schema achieves the highest Schema Quality Score (SQS), followed by SBERT-RDF and GEO-Nautilus. The comparative ordering is consistent across repeated runs and the confidence intervals, shown in Fig. 2, indicate stable behavior for all methods with negligible variance.

**Fig. 3:**
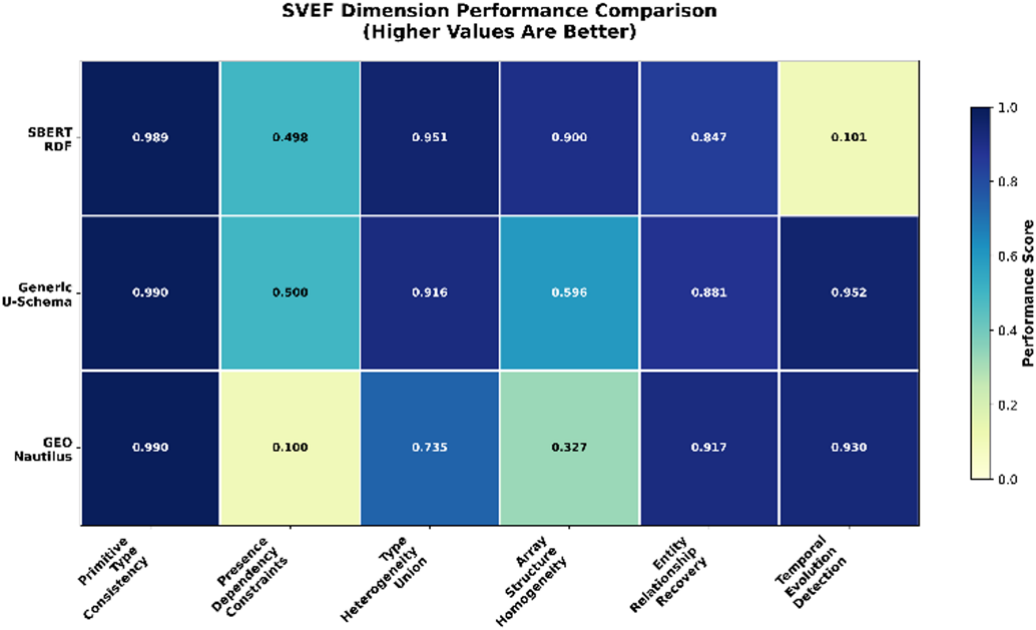
SVEF dimension performance comparison.

A more fine-grained examination of the six evaluation dimensions, as illustrated in Fig. 3, reveals substantial variation across structural, semantic, and temporal aspects of schema quality. While all approaches achieve very high Data Type Accuracy under the present benchmark conditions, more pronounced differences emerge in the higher-level dimensions. GEO-Nautilus exhibits strong performance in Entity Relationships, outperforming the alternatives in reconstructing reference structures and cardinalities. In contrast, its performance in array modelling and heterogeneous type handling is markedly lower, indicating difficulty in capturing complex nested patterns.

SBERT-RDF demonstrates a more balanced profile. It performs competitively in union modeling and array homogeneity, benefiting from semantic embedding–based generalization, yet remains limited in detecting presence dependencies and temporal drifts. Generic U-Schema shows complementary strengths: it reliably captures required/optional distinctions and demonstrates near-ideal temporal evolution detection, as reflected by its high TED score, but exhibits reduced performance when modeling heterogeneous array structures.

**Fig. 4:**
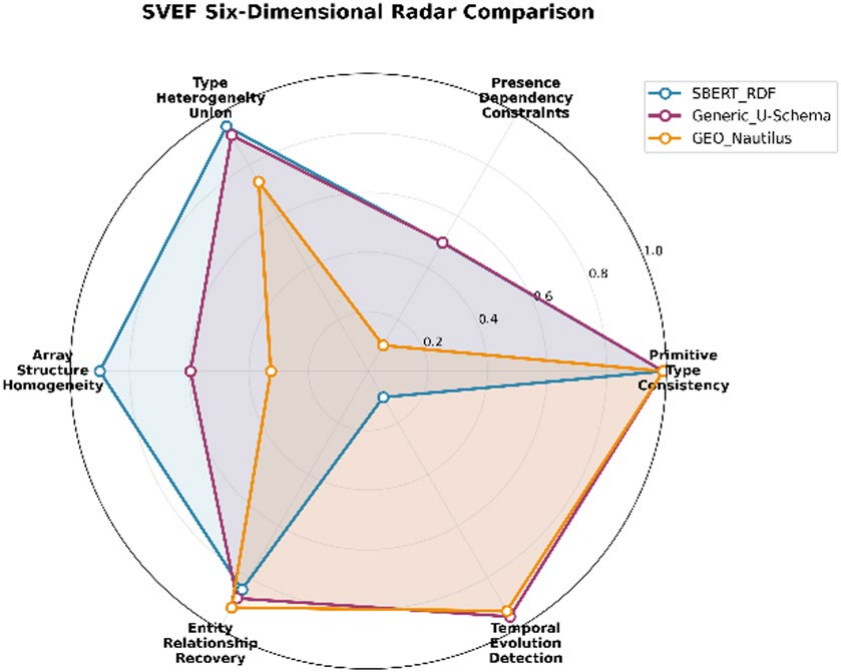
SVEF six-dimensional radar comparison.

These contrasting results underscore the importance of SVEF’s multi-dimensional evaluation strategy. No single method dominates across all dimensions and each exhibits specific strengths aligned with its underlying inference principles. The radar chart in Fig. 4 provides a consolidated view of these trade-offs, clearly illustrating how different architectural assumptions lead to distinct schema reconstruction behaviors.

Collectively, the results demonstrate that SVEF provides a sensitive and discriminative assessment across heterogeneous inference strategies. The framework not only quantifies global accuracy through SQS but also exposes structural and temporal nuances that would remain undetected under single-metric evaluation. This confirms the value of a multi-perspective validation methodology for advancing research in NoSQL schema inference.

## Discussion

The experimental evaluation provides a detailed view of how current schema extraction techniques behave when assessed through the multi-dimensional perspective of SVEF. This section interprets the broader patterns revealed by the evaluation, with an emphasis on what they indicate about the inherent challenges of inferring structure from schemaless NoSQL data. The intention is not to repeat numerical results, but to examine the tendencies, systematic gaps and characteristic strengths that emerge across methods and datasets.

By decomposing schema quality into several well defined dimensions, SVEF makes it possible to identify which aspects of structural and semantic organization are consistently recovered by existing approaches and which dimensions remain difficult to infer. This multi perspective lens also highlights the trade-offs that different techniques make across type modeling, constraint inference, array structure characterization, relationship reconstruction and temporal alignment. Situating these observations within the broader research landscape allows this discussion to clarify the conceptual insights generated by the evaluation and to prepare the ground for the practical implications and future directions that follow.

### Interpretation of method performance across dimensions

The comparative analysis across the six evaluation dimensions reveals consistent patterns in the strengths and limitations of the three examined schema extraction methods. All approaches achieve very high scores in Data Type Accuracy, suggesting that primitive type detection is handled effectively in the present controlled evaluation setting. Within these benchmark conditions, this dimension appears less discriminative than other aspects of schema inference. This stability across datasets suggests that type identification benefits from directly observable regularities in document instances and requires less inferential complexity than dimensions involving dependencies, cross-document relations, or temporal variation.

A different trend appears in Required and Optional Fields. Scores in this dimension are systematically lower for all methods, with the largest drop observed for the approach that does not model conditional or co-occurrence information. These results highlight that requiredness inference and dependency reconstruction remain highly sensitive to data sparsity, optional fields and uneven distributions of properties. They also suggest that many schema extraction techniques rely primarily on frequency thresholds, which are not always sufficient to capture meaningful structural constraints.

Performance diverges more clearly in the type heterogeneity dimension. Methods that explicitly model union types tend to approximate the observed variability more effectively, while methods with static or majority-based type assumptions under-represent genuine heterogeneity. This observation aligns with the known challenge of capturing polymorphic fields in schemaless data, where the absence of explicit type declarations places significant weight on distributional cues.

Large differences also appear in Collection Structure Consistency. Methods that apply detailed structural analysis of nested content obtain notably higher scores, whereas approaches that treat arrays as unstructured collections show reduced accuracy. This confirms that array modeling requires explicit attention to depth, nesting profiles and item regularity, since these properties vary substantially across real-world document collections.

Entity Relationships displays a complementary pattern. Techniques that maintain explicit notions of entity boundaries or exploit recurrent reference patterns recover relationships more consistently. In contrast, methods that focus primarily on document-level statistics are less successful at reconstructing links and cardinalities. This behavior reflects the fact that relationships are often implicit and require cross-document reasoning rather than local inspection.

The most pronounced contrast emerges in temporal evolution detection. Only approaches equipped with mechanisms for tracking schema changes over time achieve high scores, whereas static inference techniques yield low alignment with ground-truth evolution boundaries. This confirms that temporal schema drift is fundamentally different from structural inference and requires targeted modeling of version boundaries and transition events.

Overall, the dimension-wise interpretation shows that schema extraction methods excel in areas where structure is directly observable in isolated documents and struggle where inference depends on sparsity-aware reasoning, cross-document alignment, or temporal dynamics. This perspective clarifies the specific capabilities and blind spots of current techniques and motivates the need for more comprehensive models capable of integrating evidence across the full spectrum of schema properties.

### Comparative analysis

The comparative results reveal that each baseline approach embodies distinct methodological priorities that shape its performance across the six SVEF dimensions. SBERT RDF places emphasis on capturing semantic signals at the property and entity levels, which supports strong recovery of type variants and array structure regularities. However, its semantic alignment strategy offers limited guidance for detecting version boundaries or subtle schema drifts, which explains the relatively low scores in temporal evolution detection. Generic U-Schema, in contrast, adopts an explicit structural modeling paradigm that enables precise identification of relationship patterns and accurate detection of schema evolution events. This design yields the highest overall quality score, but its deterministic modeling of array specifications leads to weaker performance in heterogeneity-rich settings. GEO-Nautilus favors graph-based extraction with a focus on structural connectivity, which contributes to high relationship recovery scores. At the same time, the approach exhibits challenges in handling type heterogeneity and optionality, likely due to simplifications in its property aggregation mechanism.

These differences illustrate that the observed performance gaps are not incidental but trace back to each method’s modeling philosophy. Methods that prioritize semantic flexibility tend to excel in capturing heterogeneous type patterns but may struggle with evolution-aware constraints, while structurally driven approaches often recover relationships and version changes more reliably but may underfit irregular property behavior. The resulting trade-offs highlight the diversity of strategies underlying current schema inference techniques and underscore the need for multifaceted evaluation frameworks such as SVEF, which can uncover strengths that might otherwise remain hidden behind aggregate metrics.

### Cross-dataset behavior and generalization patterns

The cross-dataset analysis provides additional insight into how inference strategies respond to variations in structural complexity, heterogeneity and temporal dynamics. Despite sharing the same evaluation pipeline, the three datasets present distinct challenges: the e-commerce dataset exhibits controlled heterogeneity and explicit evolution boundaries, the healthcare dataset contains deeply nested structures with extensive type variability and the IoT dataset foregrounds temporal drift with comparatively simple structural forms. These characteristics make cross-dataset performance an informative indicator of each method’s degree of generalization.

Generic U-Schema shows the most consistent behavior across datasets, particularly in dimensions associated with structural organization and evolution tracking. This stability suggests that its modeling principles generalize well when entity relationships and version boundaries are clearly encoded, even when type distributions vary. SBERT-RDF demonstrates strong transferability for type-oriented dimensions, confirming the robustness of its semantic matching strategy in contexts where property variability is high. However, its sensitivity to dataset-specific temporal cues results in reduced stability when the evolution pattern changes. GEO-Nautilus, although strong in relationship reconstruction, exhibits more pronounced variation across datasets, especially in settings with heterogeneity or irregular optionality. This variability indicates that its graph-based extraction mechanism is effective when structural anchors are dominant but less adaptable in scenarios driven by dynamic or heterogeneous property behavior.

Overall, the cross-dataset patterns highlight that generalization is strongly linked to the methodological assumptions embedded in each approach. Methods grounded in semantic flexibility adapt well to variable attribute distributions, while those guided by explicit structural modeling maintain stability when entity interactions and version transitions are central. This reinforces the value of using multiple controlled datasets in evaluation, as it reveals performance tendencies that would remain obscured in single-dataset studies.

### Limitations and future directions

Although the evaluation provides detailed insight into comparative accuracy across the six SVEF dimensions, several limitations remain. First, the study focuses on correctness and does not systematically measure runtime efficiency, memory usage, or scalability. These factors are important in practical settings where schema extraction must operate over large and evolving document collections under constrained resources. Future work should therefore incorporate explicit measurements of execution time, peak memory usage, and scalability.

A second limitation concerns the static nature of the evaluation environment. Real-world deployments often involve continuous ingestion, partial data availability, and asynchronous schema evolution^24^. Evaluating the approaches under streaming or incremental settings would provide a clearer view of how efficiently they adapt to evolving workloads.

A third limitation concerns the use of curated benchmark datasets with fully specified reference schemas. This design supports controlled and fine-grained evaluation across all SVEF dimensions, but it may also favour approaches that align more closely with the modelling assumptions encoded in the benchmark construction process. As a result, the current evaluation may not fully reflect the irregularity, incompleteness, and noise often encountered in real-world document-oriented data. Extending the study to include real-world datasets, even where only partial gold truth is available, would strengthen the external validity of the framework.

A fourth limitation concerns the use of fixed global thresholds and weighting parameters within the evaluation framework. While this choice supports comparability across methods and datasets, the present study does not examine the sensitivity of scores or method rankings to variations in these parameters. Future work should therefore include systematic sensitivity analysis.

A further limitation concerns the scope of the temporal evaluation itself. The Temporal Evolution Detection dimension assesses version boundaries, change behaviour, and alignment with reference evolution patterns. It does not evaluate migration correctness, transformation validity, or downstream effects such as query compatibility after schema evolution. These aspects remain outside the present scope of SVEF.

Overall, future work should extend the framework toward greater computational realism, broader dataset coverage, parameter robustness, and richer operational evaluation.

## Conclusion

This work introduced the Schema Validation and Evaluation Framework (SVEF), a unified model for assessing the quality of schemas inferred from schemaless NoSQL data. The framework addresses a longstanding gap in the field by defining measurable standards of correctness that extend beyond structural coverage to include type variability, array organisation, property dependencies, inter-entity relationships and temporal evolution. Through its six complementary dimensions, SVEF provides a coherent view of schema validity that is independent of any specific inference algorithm, data model, or implementation strategy.

The empirical study applied SVEF to three representative schema extraction approaches and demonstrated its ability to reveal differences that are not captured by traditional evaluation practices. While Data Type Accuracy and Type Heterogeneity were consistently well supported across methods, the dimensions related to conditional constraints, array structure and evolution handling showed substantial variation. These findings indicate a need for more balanced inference strategies that perform reliably across all dimensions, rather than excelling in isolated aspects of schema reconstruction.

Beyond empirical insights, the results point to future opportunities for advancing schema extraction techniques, particularly through improvements in handling complex dependencies, modelling long-term evolution and managing computational efficiency for large-scale deployments. SVEF provides a structured basis for these future developments and supports continued progress toward more robust and adaptable schema inference solutions.

## Data Availability

The datasets generated and analysed during the current study are available in the GitHub repository, https://github.com/saadbelefqih/SVEF.

## Code availability

All evaluation procedures described in this manuscript are implemented in a publicly available code repository. The repository contains a single Python file, SVEF.py, which provides the complete implementation of the proposed schema evaluation framework, including metric definitions and computation routines for all evaluation dimensions. The code enables reproducible evaluation of inferred schemas based on dataset-derived structural, semantic, and temporal properties. The repository is available at: https://github.com/saadbelefqih/SVEF
